# Control of Transcription by Cell Size

**DOI:** 10.1371/journal.pbio.1000523

**Published:** 2010-11-02

**Authors:** Chia-Yung Wu, P. Alexander Rolfe, David K. Gifford, Gerald R. Fink

**Affiliations:** 1 Department of Biology, Massachusetts Institute of Technology, Cambridge, Massachusetts, United States of America; 2 Whitehead Institute for Biomedical Research, Cambridge, Massachusetts, United States of America; 3 Computer Science and Artificial Intelligence Laboratory, Department of Electrical Engineering and Computer Science, Massachusetts Institute of Technology, Cambridge, Massachusetts, United States of America; 4 Broad Institute of MIT and Harvard, Cambridge, Massachusetts, United States of America; Harvard Medical School, United States of America

## Abstract

Cell size increases significantly with increasing ploidy. Differences in cell size and ploidy are associated with alterations in gene expression, although no direct connection has been made between cell size and transcription. Here we show that ploidy-associated changes in gene expression reflect transcriptional adjustment to a larger cell size, implicating cellular geometry as a key parameter in gene regulation. Using RNA-seq, we identified genes whose expression was altered in a tetraploid as compared with the isogenic haploid. A significant fraction of these genes encode cell surface proteins, suggesting an effect of the enlarged cell size on the differential regulation of these genes. To test this hypothesis, we examined expression of these genes in haploid mutants that also produce enlarged size. Surprisingly, many genes differentially regulated in the tetraploid are identically regulated in the enlarged haploids, and the magnitude of change in gene expression correlates with the degree of size enlargement. These results indicate a causal relationship between cell size and transcription, with a size-sensing mechanism that alters transcription in response to size. The genes responding to cell size are enriched for those regulated by two mitogen-activated protein kinase pathways, and components in those pathways were found to mediate size-dependent gene regulation. Transcriptional adjustment to enlarged cell size could underlie other cellular changes associated with polyploidy. The causal relationship between cell size and transcription suggests that cell size homeostasis serves a regulatory role in transcriptome maintenance.

## Abstract 2

The demonstration of a causal, regulatory relationship between cell size and gene expression in yeast suggests that cells maintain size in order to maintain transcriptional homeostasis.

## Author Summary

Cells of the same type, whether microbial, plant, or metazoan in origin, exhibit remarkable uniformity in size. This uniformity arises from control mechanisms that respond to internal cellular changes as well as external environmental factors. Although precise control of cell size is a universal phenomenon, its relationship to cellular physiology is underexplored. In this study using yeast we show a causal relationship between cell size and gene regulation: changes in cell size correlate with changes in the expression of a set of genes. Hence, the maintenance of uniformity in cell size could be a homeostatic mechanism for the maintenance of gene expression in a cell or in a population of cells within a tissue. The relationship between cell size and gene expression uncovered in this study may have fundamental implications in evolution, in the development of multicellular organisms, and in the formation of tumors, as these processes often involve genome duplication accompanied by enlarged cell size.

## Introduction

The size of cells can vary significantly within an organism, and cells of the same type display pronounced increase in size with increasing ploidy [1],[2]. During development, specific cell types in many diploid organisms perform endoreplication and differentiate into polyploid cells that are functionally distinct from their diploid progenitors [2]. Polyploidy also occurs as an intermediate state in aneuploid tumor formation [3] and as a mechanism to create substrates for evolution [4],[5]. From yeast to mammals, polyploidy is associated with enlarged cell size and altered cellular physiology [2],[6]–[8]. How polyploidy changes physiology is a long-standing question. Furthermore, a causal relationship between enlarged cell size and altered physiology has not been discovered.

Yeast offers a unique advantage in studying the physiological consequences of polyploidy, because it is possible to construct isogenic strains of increasing ploidy. There were two previous analyses that compared transcription between cells of different ploidy. The first analysis of transcription in a yeast ploidy series identified a few genes whose transcript abundance in the transcriptome was altered by ploidy [6]. These included some genes that were strongly repressed and others that were strongly induced in polyploids. Although this study established a clear effect of ploidy on transcription, the limited set of identified genes did not reveal a functional relationship between ploidy and gene expression. The scope and sensitivity of this early investigation were hampered by technical limitations. Because the genome sequence of the studied yeast strain (Σ1278b) was not known at the time, microarrays designed for a related yeast strain (S288c) were employed. Recent genome analysis comparing these two yeast strain backgrounds has revealed many polymorphisms and changes in genomic organization [9] that compromised the power of detection by hybridization in the earlier study.

A subsequent analysis of polyploid yeast detected no significant differences between the diploid and tetraploid transcriptomes by microarrays [8], raising the possibility that the differences found in the first study were strain specific. Alternatively, experimental differences between the two studies could account for the different conclusions. Expanding the second study to compare strains with a greater difference in ploidy (i.e., between haploids and tetraploids) might have uncovered significant transcriptional changes related to ploidy, as was observed in the first study. More importantly, this later study used a different laboratory strain (S288c). Unlike the strain used in the first study (Σ1278b), the S288c strain background does not express *FLO11* [10], the gene that was most affected by ploidy in the first study [6].

We address the issues raised by both of these studies by examining ploidy effects in different yeast strains with more sensitive assays. The recently acquired genome sequence of the Σ1278b strain [9], combined with advances in transcriptome profiling by RNA-seq [11], provides the resolution necessary for genome-wide determination of a functional connection among genes regulated by ploidy. The dynamic range of quantitative linearity in RNA-seq is at least 10-fold higher than that of microarrays, making RNA-seq superior at comparing transcript abundance [12]. In this study, RNA-seq enabled identification of a much larger set of differentially expressed genes between Σ1278b haploid and tetraploid strains and the discovery of related genes by Gene Ontology (GO) analysis. The enriched GO terms suggested a causal relationship between cell size and gene expression, and this relationship was then confirmed by analyzing gene expression patterns in cells of varying sizes. The genes repressed in large cells of the Σ1278b background were also found to be repressed in tetraploids of S288c, suggesting that the casual relationship between cell size and gene expression is a general feature.

## Results

### RNA-Seq Reveals Novel Genes Differentially Regulated in Tetraploids as Compared with Haploids

**Figure 1:**
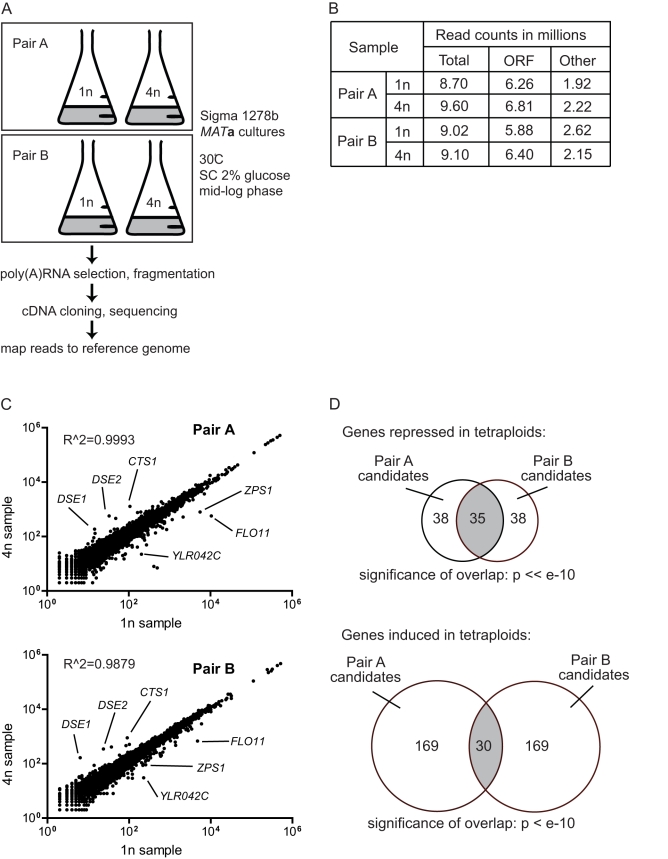
Identification of ploidy-regulated genes in yeast (Σ1278b). (A) RNA-seq analysis of the Σ1278b haploid and tetraploid transcriptomes. Two pairs of haploid and tetraploid cultures, A and B, were processed for transcriptional profiling (using Illumina Genome Analyzer 2). Reads were mapped to the annotated Σ1278b genome. We calculated ORF expression based on the number of reads mapping inside the ORF. SC, synthetic complete medium. (B) RNA-seq read counts. More than 90% of the approximately 9 million reads in each sample were mapped to the genome. More than 60% of the mapped reads fell within annotated ORFs. The majority of remaining reads mapped to the rDNA locus and Ty elements. The processed data (with calculations for fold changes and *p*-values) are in Dataset S1. (C) Ploidy alters expression of only a small number of genes. Read counts for all transcripts at the two ploidies are plotted, and several differentially regulated genes are specified. The comparable expression of most transcripts between the two ploidies shows that the tetraploid cells were euploid [42]. (D) Identification of genes differentially expressed in the tetraploid. Within each pair of haploid (1*n*)–tetraploid (4*n*) RNA-seq datasets, differentially expressed candidate genes were ranked by fold change in expression. Comparison of top-ranking candidates between both pairs produced overlapping genes that were called differentially expressed (see details in Materials and Methods). Cutoffs for top-ranking candidates were selected to obtain a sufficient set of overlapping genes for GO analysis while ensuring that the overlap remained highly statistically significant by hypergeomtric test.

**Figure 2:**
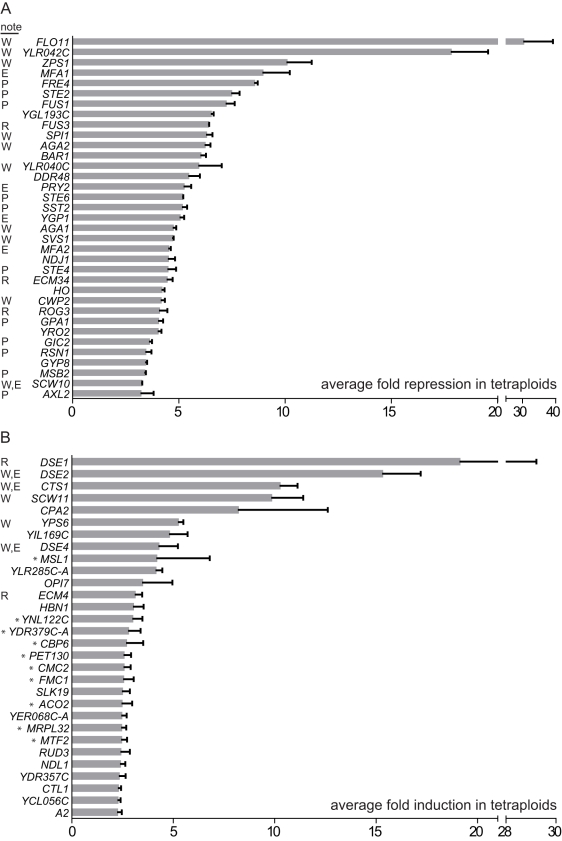
Genes differentially regulated in the Σ1278b tetraploid and their compartmental bias for encoding cell surface components. Differentially expressed genes in the Σ1278b tetraploid are ranked by average fold change in expression from both pairs of RNA-seq data. Error bar indicates standard deviation. (A) Genes repressed in the tetraploid. (B) Genes induced in the tetraploid. Characterized genes are shown with their standard names in SGD. Notes on localization of the encoded protein according to SGD: E, extracellular space; P, plasma membrane; R, regulator of cell surface components with intracellular or unknown localization; W, cell wall. Asterisks indicate mitochondrial localization; this category is not statistically significant in our GO analysis but nevertheless represents a third of the induced genes. We did not find a bias for specific cell cycle stages among the differentially regulated genes (Table S1) [43].

To identify transcripts whose relative abundance in the transcriptome is changed by ploidy, poly(A) RNA transcripts isolated from isogenic haploids and tetraploids of the Σ1278b strain background were analyzed by RNA-seq (Figure 1A). Approximately 9 million sequence reads were obtained from each cDNA library, and the majority of reads mapped to annotated ORFs (Figure 1B). Pair-wise comparison of tetraploid samples with haploid samples revealed that ploidy affects the abundance of only a small proportion of the total transcripts (Figure 1C). By comparison with haploids, 35 transcripts were significantly and reproducibly repressed and 30 transcripts were induced in tetraploids (Figure 1D). The differentially expressed genes included several of the strongly regulated genes identified in the previous study on a Σ1278b ploidy series, and the majority of the remaining genes showed consistent regulatory trends in both studies (Figure 2; Dataset S1) [6]. The disproportional expression of these genes appeared unrelated to the cell cycle, since there was no systematic bias for genes expressed in specific cell cycle stages (Table S1).

### Gene Ontology Analysis Suggests That Cell Size but Not Ploidy Alters Gene Expression in Tetraploids

Interestingly, the genes differentially expressed in tetraploid cells are significantly enriched not for those associated with chromosomes but for those encoding proteins localized to the cell surface (cell wall, extracellular space, and plasma membrane) and for genes that encode regulators of cell surface components (Figure 2; Tables 1 and S2, S3, S4, S5). This compartmental bias suggests that the differential gene expression in tetraploids is not directly caused by an increase in the genome content, but by a difference in cell size/geometry: for a spherical cell, a 4-fold increase in volume corresponds to only a ∼2.5-fold increase in surface area. In other words, although tetraploid yeast cells are 4-fold larger in volume than haploid cells [13], the ratio of surface area to volume is smaller in tetraploids than in haploids. Reduction in surface area relative to volume is likely to trigger differential regulation of components associated with the cell surface, where signaling and transport processes take place dynamically. A reduction in relative cell surface area could alter interactions between surface and cytoplasmic signaling pathway components and affect the cell's ability to transport metabolites across the plasma membrane. Either type of perturbation caused by a reduced surface area relative to volume could alter gene expression in enlarged cells.

**Table 1:** Cellular compartmental Gene Ontology terms for genes differentially regulated in the Σ1278b tetraploid.

GO Term	Cluster Frequency	Background Frequency	*p*-Value
Cell wall	15/65 (23.1%)	85/5,613 (1.5%)	1.5 e^−14^
Extracellular	8/65 (12.3%)	22/5,613 (3.9%)	5.8 e^−11^
Plasma membrane	11/65 (16.9%)	254/5,613 (4.5%)	1.1 e^−4^

### Haploid Size Mutants Also Demonstrate Regulation of Gene Expression by Size

**Figure 3:**
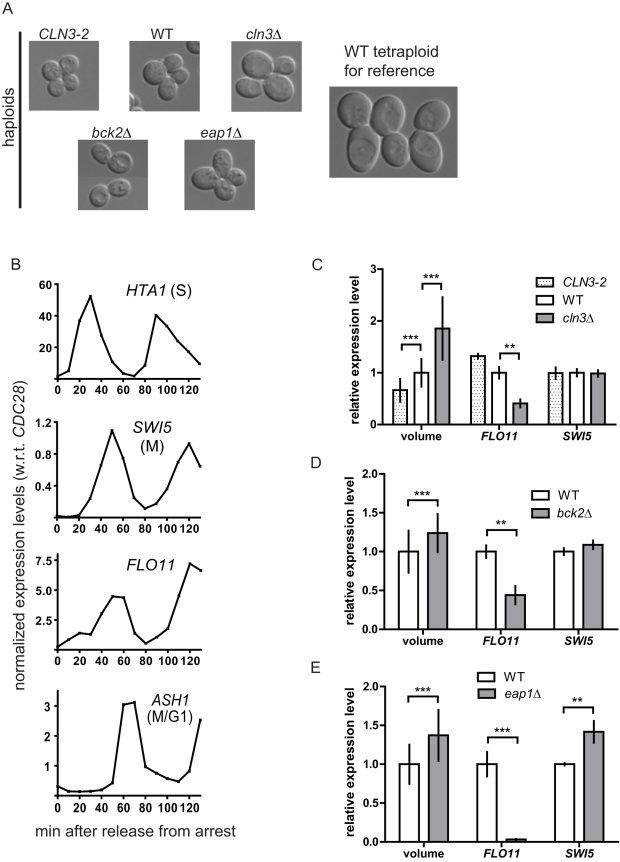
Enlarged cell size represses *FLO11*. (A) Live cell images of WT and size mutant haploids. (B) *FLO11* transcript abundance peaks in M-phase during the mitotic cycle. Upon release from alpha-factor arrest in G1, WT haploid cells were harvested at 10-min intervals. Cell cycle stages were assessed using expression profiles of known standard transcripts: *HTA1* (S-phase), *SWI5* (M-phase), and *ASH1* (M/G1 transition) [43]. The expression pattern of *FLO11* resembles that of *SWI5*. It is worth noting that *FLO11* was not found to be regulated by the cell cycle in a previous genome-wide study, because of a difference in yeast strain background (W303) [10],[43]. (C–E) Abundance of the *FLO11* transcript inversely correlates with cell size in haploids. (C) *CLN3*-based size series (*MAT*alpha strains). (D) WT and *bck2*Δ *MAT* **a** strains. (E) WT and *eap1*Δ *MAT* **a** strains. Cln3 is the most upstream activator of G1/S transition and maintains the size threshold of mitotic START [1]. Cln3 also regulates vacuolar morphology [29],[30]. Bck2 promotes G1/S transition independently of Cln3 [44]. Eap1 regulates translation [31] and has a separate role in chromosome segregation [32]. Cell volume was measured from microscopy images. Gene expression was measured by qPCR. Expression of *SWI5* was monitored to rule out the effect of cell cycle in data interpretation. Cell cycle arrest efficiency with nocodazole (>70%) was assessed by counting the percentage of cells arrested in metaphase (Table S6). Error bars indicate standard deviation. Statistical significance was calculated using Student's *t* test. *, *p*<0.05; **, *p*<0.01; ***, *p*<0.001. *n* = 50 for volume measurements. *n* = 3 for transcript quantification.

The relationship between cell size and transcription could be assessed by examining gene expression levels in haploid mutants with altered cell size relative to wild type (WT). Cell size mutants (Figure 3A) previously identified in a genome-wide study [14] were selected because they effectively enlarge cell size without significantly affecting fitness or cell shape. In addition, the underlying mutations have no reported functional relationship with the differentially expressed genes identified in tetraploid cells. We considered additional mutations to increase cell size in haploids [15],[16] but did not pursue them because of technical concerns that would preclude a clear interpretation of experimental results (Text S1).

If differential expression of a gene in tetraploids is caused by enlarged cell size rather than directly by higher ploidy, the gene should be differentially expressed in haploid size mutants as well. Furthermore, the magnitude of change in the gene's expression level should, ideally, correspond to the magnitude of change in cell size. This cell size–transcription hypothesis was initially tested by investigating the effect of cell size on expression of *FLO11*, a gene encoding a cell surface glycoprotein [17],[18]. *FLO11* showed the highest degree of repression in the tetraploid (Figure 2A), providing a wide range of detection for changes in transcript levels.

Expression levels of *FLO11* were measured in WT Σ1278b and isogenic size mutant haploids (Figure 3A) treated with nocodazole for cell cycle arrest in M-phase, when the *FLO11* transcript is most abundant (Figure 3B). Because the size mutants manifest an altered cell cycle [1],[14], arresting the cell cycle was necessary to separate the transcriptional effect of cell size from that of cell cycle. We analyzed mutant alleles of the *CLN3* gene that altered cell size significantly and arrested efficiently in the presence of nocodazole. The *CLN3-2* mutant arrested as small cells (66% of WT volume), whereas the *cln3*Δ mutant arrested as large cells (185% of WT volume) (Figure 3C). In this haploid size series, we found an inverse correlation between *FLO11* expression and cell size: *FLO11* transcript abundance is highest in the small *CLN3-2* haploid and lowest in the large *cln3*Δ haploid, the same relationship observed between *FLO11* expression and cell size in haploid versus tetraploid cells. Expression of *FLO11* was also significantly repressed in the *bck2*Δ and *eap1*Δ mutants that displayed enlarged cell size at 124% and 137% of WT volume, respectively (Figure 3D and 3E). The reduced expression of *FLO11* in these large mutants mimics the down-regulation of *FLO11* in tetraploids (Figure 2A). The results from the haploid size mutants demonstrate an inverse correlation between *FLO11* expression and cell size, and this correlation is independent of ploidy.

To see whether other genes differentially expressed in tetraploid cells were also influenced by cell size, we used quantitative PCR (qPCR) to compare expression of these genes in WT and *cln3*Δ haploids. Among the mutants we examined, the *cln3*Δ haploid displayed the most pronounced change in cell size and arrested in M-phase with efficiency most similar to WT (Figure 3; Table S6). The majority of differentially regulated genes in the tetraploid were regulated in the same direction in the *cln3*Δ haploid (Tables 2, S7, and S8), especially the top-ranking genes, i.e., those that displayed the strongest differential expression in tetraploids (Figure 2A and 2B). These top-ranking genes likely represent those that respond most robustly to changes in cell size, since the *cln3*Δ haploid (185% of WT haploid volume) is still much smaller than the tetraploid (400% of WT haploid volume). Notably, the fold changes in expression levels of the top-ranking genes appeared to correlate with the increase in cell size: they were smaller in the *cln3*Δ haploid and larger in the WT tetraploid (Tables S7 and S8), a trend consistent with a functional relationship between cell size and gene expression.

**Table 2:** A substantial number of differentially expressed genes in the tetraploid are also differentially expressed in the *cln3*Δ haploid.

Genes Repressed in Tetraploid	Genes Induced in Tetraploid
$ *FLO11*	$ *DSE1*
$ *YLR042C*	$ *DSE2*
$ *MFA1*	$ *CTS1*
$ *FRE4*	$ *SCW11*
$ *STE2*	$ *CPA2*
$ *FUS1*	$ *YPS6*
$ *FUS3*	$ *YIL169C*
$ *AGA2*	*DSE4*
$ *BAR1*	*MSL1*
$ *YLR040C*	*OPI7*
$ *DDR48*	$ *ECM4*
$ *PRY2*	$ *HBN1*
$ *STE6*	*YNL122C*
$ *SST2*	*YDR379C-A*
*AGA1*	*CBP6*
$ *SVS1*	**∧** *CMC2*
$ *MFA2*	*FMC1*
$ *NDJ1*	*SLK19*
$ *STE4*	*ACO2*
*HO*	*MRPL32*
$ *CWP2*	*MTF2*
*GPA1*	*RUD3*
$ *GIC2*	*NDL1*
$ *RSN1*	*YDR357C*
*GYP8*	*CTL1*
*MSB2*	
*SCW10*	

Among the genes differentially expressed in the Σ1278b tetraploid, as identified from the RNA-seq experiment (Figure 2A and 2B), listed here are 52 genes that remain expressed and differentially regulated in tetraploid cells cultured in the YPD growth medium. WT and *cln3*Δ haploids were cultured in YPD plus nocodazole, and equivalent cell cycle arrest was monitored as described in Figure 3. Expression levels were measured by qPCR and analyzed with Student's *t* test (*n* = 3). Genes expressed at significantly different levels (*p*<0.05) are labeled as follows: $, genes regulated in the same trend in the *cln3*Δ haploid and the WT tetraploid; ∧, gene showing the opposite trend. Quantitative data for gene expression levels and fold changes are summarized in Tables S7 and S8.

**Figure 4:**
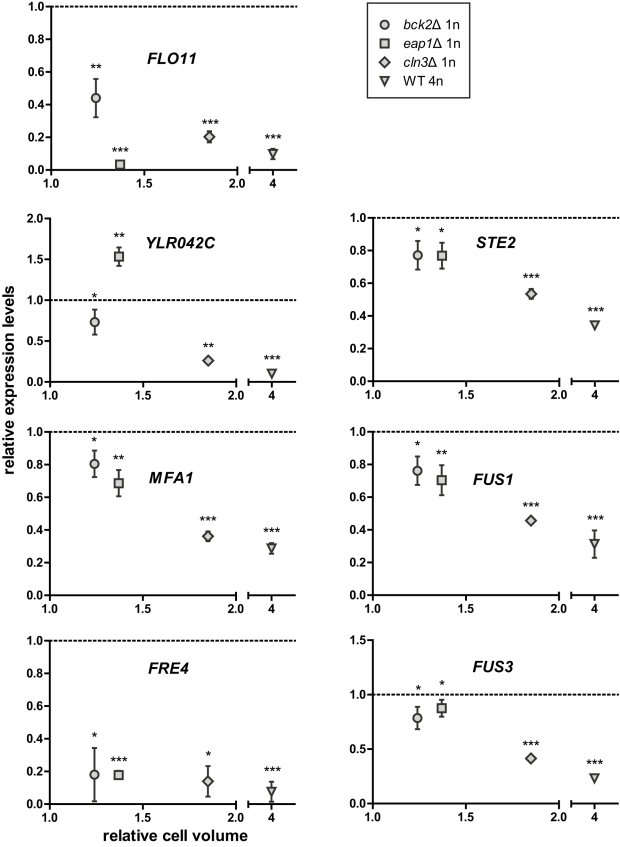
Differential regulation of the top-ranking size-repressed genes correlates with cell size. Gene expression and cell size in each of the enlarged strains were compared to an isogenic WT haploid. The dashed line represents the expression level in the WT haploid, which was set to 1. Asterisks above each symbol denote the significance of difference in transcript level (*, *p*<0.05; **, *p*<0.01; ***, *p*<0.001; error bars indicate standard deviation; statistical significance was calculated using Student's *t* test with *n* = 3). To separate the effects of cell size from cell cycle, the comparison between WT and mutant haploids required cells treated with nocodazole, whereas the comparison between haploid and tetraploid required asynchronously grown cells (Table S7B). The expression levels of *FLO11* and *YLR042C* in the *eap1*Δ mutant appeared significantly different from those in other size mutants. This mutation likely caused additional perturbations in the cell besides increasing cell size, and these perturbations may have specific effects on *FLO11* and *YLR042C*.

**Figure 5:**
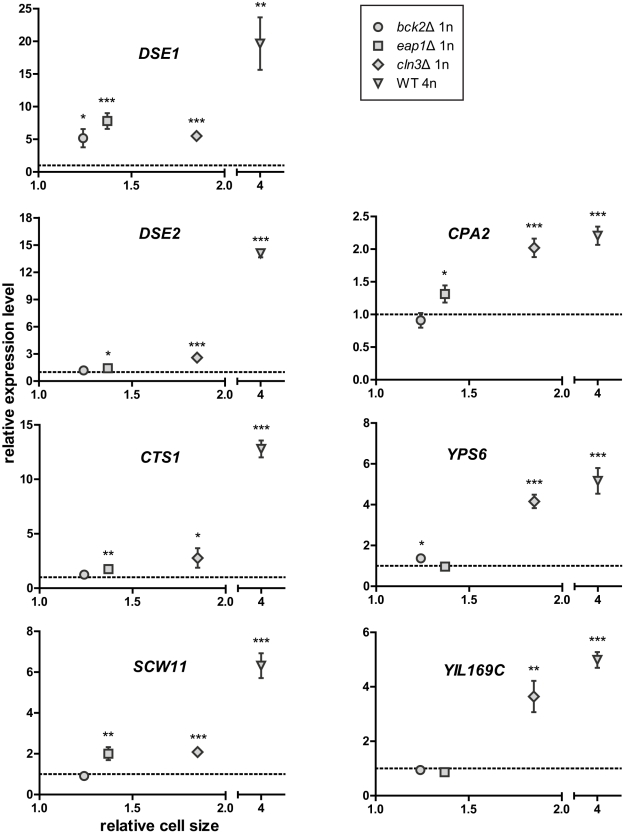
Differential regulation of the top-ranking size-induced genes correlates with cell size. See legend for Figure 4.

To determine whether the magnitude of change in transcription correlates with the magnitude of change in cell size, we compared expression of the top-ranking size-responsive genes in enlarged haploid mutants and the WT tetraploid. Expression levels in each enlarged strain were measured by qPCR and normalized to those in an isogenic WT haploid. The juxtaposed datasets show a negative correlation (for repressed genes) or a positive correlation (for induced genes) between gene expression levels and cell size (Figures 4 and 5; Table S9). The results indicate that the differential regulation of these genes is enhanced with increasing cell size. This observation strongly supports our cell size–transcription hypothesis and the idea that incremental changes in cell size can be sensed by the cell and lead to incremental transcriptional responses.

### Gene Ontology and Transcription Factor Motifs Reveal Pathways That Mediate Size-Dependent Gene Regulation

GO analysis indicated that many of the genes repressed by large cell size are regulated by the mating and the filamentation mitogen-activated protein kinase (MAPK) pathways (Table S3; see complete GO analysis results in Dataset S1). Analysis of transcription factor binding motifs also suggested that these MAPK pathways mediate differential gene regulation in response to cell size: the binding motifs of Dig1 and Ste12, transcription factors that function in both pathways, were significantly enriched (Table S10; Dataset S2). Ste12 is a transcriptional activator crucial for mating and filamentation [19]. Dig1 mediates transcriptional repression in both cellular processes by inhibiting the activity of Ste12 [20],[21]. When either of the MAPK pathways is active, Ste12 is phosphorylated by the MAPK and released from inhibition by Dig1.

**Figure 6:**
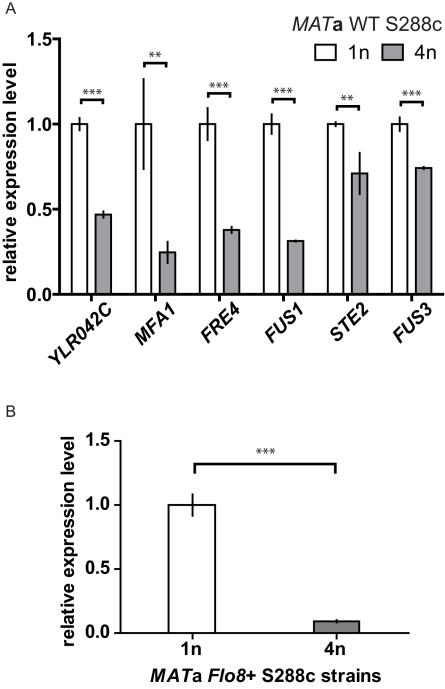
Differential down-regulation of the top-ranking size-repressed genes also occurs in the S288c strain background. (A) Expression levels of the top-ranking size-repressed genes were examined in the WT *MAT* **a** haploids and tetraploids of the S288c strain background. *MFA1*, *FUS1*, *STE2*, and *FUS3* are genes regulated by the mating MAPK pathway. The function of *YLR042C* is unknown, but the presence of Dig1 and Ste12 motifs in its promoter (Table S10) suggests that it could be regulated by the mating pathway. *FRE4*, not involved in the mating response, was included in the analysis because it is one of the most size-regulated genes in Σ1278b. *FLO11* is not expressed in this background [10] and thus was omitted. (B) Transcript levels of *FLO11* in S288c strains expressing functional Flo8, a transcriptional activator required for *FLO11* expression [10]. All strains were cultured asynchronously in YPD until mid-log phase, and transcript abundance was measured by qPCR. Error bars indicate standard deviation. Statistical significance was calculated using Student's *t* test with *n* = 3. **, *p*<0.01; ***, *p*<0.001.

We compared transcription in haploid and tetraploid cells of a different strain background (S288c) to see whether the mating pathway, which is conserved among different species of yeast [22], was affected by cell size in this background as it is in Σ1278b. The genes downstream of the mating pathway were differentially repressed in the S288c tetraploid (Figure 6A), suggesting that the effect of cell size on the mating pathway could be a general characteristic in yeast. We also constructed isogenic haploid and tetraploid S288c cells expressing *FLO11* [10] and found that expression of *FLO11* was significantly repressed in this tetraploid strain (Figure 6B), a result consistent with our finding in Σ1278b.

Although genes up-regulated in large cells were significantly enriched for those containing the binding motifs of Ace2, Swi5, Rfx1, and Yap7 in their promoters (Dataset S2), this group gave no obvious clues concerning the molecular pathway causing their differential regulation (Text S2). Moreover, when the transcription of this group of genes in haploids and tetraploids of the S288c background was compared, there was no difference in the levels of their expression (data not shown). This difference between the two yeast strains probably reflects the many regulatory differences between them [9] (Text S2).

### The Mating and the Filamentation Pathways Contribute to Differential Gene Expression in Response to Changes in Cell Size

**Figure 7:**
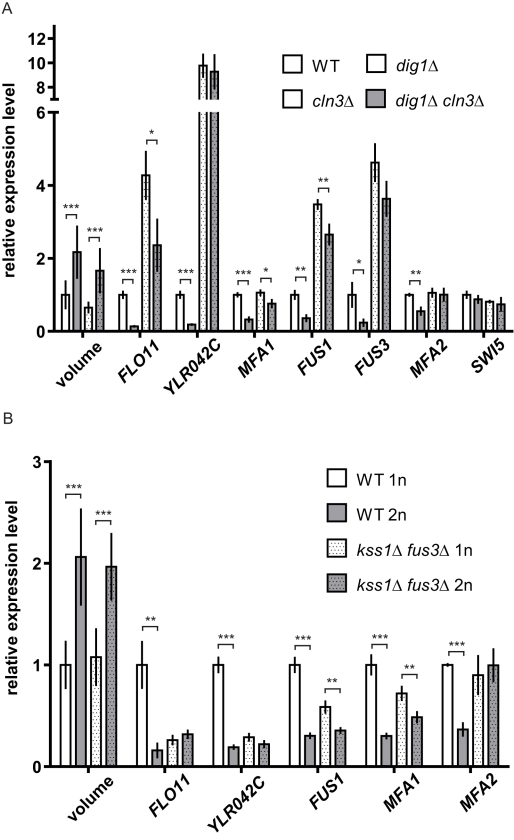
Cell size alters gene expression through the mating and the filamentation pathways. (A) The transcriptional repressor Dig1 in the mating and the filamentation pathway is involved in gene repression by cell size. In the absence of Dig1, several size-repressed genes became less responsive to an increase in cell size. The fold changes in expression between the *dig1*Δ and the *dig1*Δ *cln3*Δ mutants were smaller than those between the WT and the *cln3*Δ mutant. All strains were haploid and *MAT* **a** in the Σ1278b background. As described in Figure 3, cells were cultured in the presence of nocodazole prior to subsequent processing, and *SWI5* transcript was quantified to verify comparable cell cycle arrest. Error bars indicate standard deviation. Statistical significance was calculated using Student's *t* test; *n* = 50 for volume measurements; *n* = 3 for transcript quantification. *, *p*<0.05; **, *p*<0.01; ***, *p*<0.001. (B) The mating and the filamentation MAPKs (Fus3 and Kss1) contribute to differential regulation of downstream genes in large cells. In the *kss1*Δ *fus3*Δ double mutants, the effect of enlarged cell size on gene expression was reduced. The fold changes in expression between the mutant haploid and diploid strains were smaller than those between the WT haploid and diploid strains. Although all genes showed a reduction in fold change between the mutant haploids and diploids, expression levels of individual genes were affected differently by the double mutation. The unique response of each gene likely reflects gene-specific, additive effects of the single mutations. All strains were *MAT* **a** Σ1278b and were cultured asynchronously in YPD until mid-log phase. Statistical treatment was performed as in (A).

To understand the roles of the mating and the filamentation MAPK pathways in mediating size-dependent gene regulation, we disrupted signaling in these pathways in enlarged cells by making mutations in key pathway components. In the absence of the transcriptional repressor Dig1, several size-repressed genes were less repressed in large cells and became insensitive or much less responsive to size enlargement (Figure 7A). The reduced effect of cell size on gene expression in the absence of Dig1 shows that this transcription factor is involved in gene regulation by cell size. Assessment of the role of the MAPK pathways required a double mutant lacking both Fus3 and Kss1, MAPKs of the mating pathway and the filamentation pathway, respectively. The double mutant was necessary as these MAPKs have partially overlapping functions as transcriptional repressors [23]. The *kss1*Δ *fus3*Δ double mutation reduced the effect of enlarged cell size on the transcription of downstream genes (Figure 7B). Results from the *dig1*Δ and *kss1*Δ *fus3*Δ mutants suggest that reduced activities in the mating and the filamentation pathways contribute to differential gene regulation in large cells.

## Discussion

Cell size homeostasis has been intensively studied and shown to be controlled by a complex coordination of cell growth and cell division [1],[14],[24]–[28]. However, the functional significance of this intricate and efficient maintenance of cell size has not been addressed. Moreover, when the genome is duplicated, as in polyploids, the accompanying increase in cell size is maintained upon cell division. Such whole genome duplication has been invoked to explain evolution, development, and diseases [2]–[5], with little attention to the transcriptional consequences of the enlarged cell size that accompanies polyploidy.

To explore the functional relationship between enlarged cell size and gene expression, we first profiled the transcriptomes of isogenic Σ1278b haploid and tetraploid strains by RNA-seq. This strategy revealed a more complete catalog of the genes influenced by ploidy than was possible to achieve in a previous study [6]. Genes encoding cell-surface-related proteins were overrepresented, suggesting an effect of cell size on gene expression. The top-ranking genes whose transcription was down-regulated by an increase in genome size were also down-regulated in another laboratory strain, S288c, that has significant physiological differences from Σ1278b [9]. The fact that these genes behaved similarly in two different strains supported the hypothesis that cell size was responsible for the transcriptional effects. This cell size–transcription hypothesis was plausible because the volume of yeast cells increases proportionally with ploidy [13]. Moreover, as the cell surface area relative to cell volume decreases with increasing cell size, the reduction in cell surface area could alter gene expression by affecting cell-surface-related signaling molecules and impairing molecular transport across the plasma membrane.

The cell size–transcription hypothesis was supported by an independent assessment that measured gene expression in haploid mutants that make large cells. These large haploid mutants also showed a causal relationship between cell size and gene transcription, indicating the existence of a size-sensing mechanism that alters transcription independently of ploidy. An alternative model, in which polyploidy and size-altering mutations change transcription of size-regulated genes, whose altered expression then enlarges cell size, is unlikely. The set of size-regulated genes we identified do not themselves regulate cell size [14]. Moreover, the haploid size mutations and tetraploidy enlarge cell size by different mechanisms [1],[13] and affect different cellular processes [29]–[32]. The similar transcriptional effects observed in these physiologically different contexts of increased cell size and the enhancing of transcriptional changes with increasing cell size support our hypothesis that cell size sensing is involved in differential transcriptional regulation.

Our analysis of the transcriptional data suggests that the cell size signal may be transmitted by the mating and the filamentation MAPK pathways. Genes regulated by these pathways in Σ1278b were preferentially down-regulated in large cells and composed the most significant category in GO analysis. A previous study did not detect differential regulation of these genes, as the strains employed (*MAT* **a**/alpha S288c) were inactive for mating and filamentation [8]. We showed that in S288c strains of a suitable mating type and with a greater difference in ploidy, genes downstream of the mating pathway were also repressed. Genetic disruptions in the mating and the filamentation MAPKs as well as their common downstream transcriptional repressor *DIG1* reduced the effect of cell size on target gene expression. These results confirmed a decrease in activities of the mating and the filamentation pathways in large cells. The switch-like dual functions of the MAPKs [23],[33] and the positive feedback loops involving downstream transcription factors [34]–[37] likely exacerbate differences in pathway activity between the active state (in small cells) and the inactive state (in large cells). These attributes of the pathways may account for a nonlinear transcriptional response to changes in cell size. Consequently, the magnitude of change in gene expression did not appear to correlate with cell size or cell surface area in a simple fashion (Table S9).

Although the exact signal that initiates a size-dependent change in transcription is not known, these MAPK pathways have an architecture that is well suited to transmitting a signal from the cell surface to the nucleus. In both MAPK pathways, plasma-membrane-bound G proteins recruit and activate the MAPKKK upon stimulation by the mating pheromone or by nutrient starvation. Through a series of further protein–protein interactions, the MAPKKK in turn activates downstream kinases including the MAPK, which then translocates from the cellular periphery to the nucleus to induce gene expression [38]. An enlarged cell size could affect one or more of the molecular events in the process of pathway activation. Because the nuclear size is proportional to overall cell size in yeast [39],[40], both the nuclear surface and the cell surface experience a reduction in area relative to the enclosed volume in large cells. The reduced relative nuclear surface area could impair translocation of the MAPKs. The reduced relative cell surface area could affect the interactions between plasma-membrane-bound and cytoplasmic components in the pathway.

In summary, we showed that polyploidy-associated differential gene regulation is largely caused by an increase in cell size. The newly uncovered regulatory relationship between cell size and gene expression suggests that the uniformity of cell size in unicellular organisms and within tissues in multicellular organisms could be necessary to maintain the homeostasis of transcription. Our finding also suggests that cells monitor their geometric properties (i.e., size and shape) and adjust transcription accordingly. These physical features have not been typically considered a regulatory factor in cellular biology, especially in gene expression studies. In metazoans, the control of gene expression by cell size could contribute to the altered development of large or polyploid cells in normal tissues or to the aberrant physiology of tumor cells.

## Materials and Methods

### Yeast Growth Conditions

Yeast strains used in this study are listed in Table 3. Strains L6437 (WT *MAT* **a** haploid) and L6440 (WT *MAT* **a** tetraploid) were grown in synthetic complete medium plus 2% glucose at 30°C until mid-log phase for transcriptome profiling by RNA-seq. Cells were handled with caution to minimize passaging in order to avoid aneuploidy in the tetraploid. For cell cycle arrest using nocodazole, cultures were inoculated at low density in yeast extract peptone dextrose (YPD) plus 1% DMSO from overnight precultures and incubated at 30°C. After a few hours, the cultures in exponential phase were diluted to ∼0.15 O.D.600 in prewarmed fresh medium and incubated for another 30 min. Nocodazole was added to a final concentration of 15 µg/ml to arrest cell cycle for 3 h. Enrichment of cells in M-phase was monitored by *SWI5* RNA transcript abundance and by counting the percentage of large budded cells with DAPI-stained nuclei at the mother–bud junction. To compare gene expression levels in isogenic strains at different ploidies, cells were cultured in YPD at 30°C until mid-log phase. Cells were collected by centrifugation for RNA extraction and microscopy.

**Table 3:** Yeast strains used in this study.

Strain ID	Genotype/Description	Source
L6437	*MAT* **a** WT	Gerald Fink lab
L6440	*MAT* **aaaa** isogenic to L6437	Gerald Fink lab
yCW478	*MAT* **a** WT, *can1*Δ::*STE2*pr-*SpHIS5*, *lyp1*Δ::*STE3*pr-*LEU2*	Charles Boone lab
yCW784	yCW478 with *bck2*::kanMX	Charles Boone lab
yCW802	yCW478 with *eap1*::kanMX	Charles Boone lab
L7613	*MAT*alpha *CLN3*, *leu2*::*hisG*::*LEU2*	Gerald Fink lab
L7641	*MAT*alpha *cln3Δ*::*LEU2*	Gerald Fink lab
L7646	*MAT*alpha *CLN3-2*	Gerald Fink lab
L7609	L6437 with *cln3Δ*::*LEU2*	Gerald Fink lab
L7618	L6437 with *leu2*::his*G*::*LEU2*	Gerald Fink lab
yCW763	*MAT* **a** *dig1Δ*::kanMX	This study
yCW764	*MAT* **a** *dig1Δ*::kanMX, *cln3Δ*::*LEU2*	This study
L5486	*MAT* **a** WT, *HIS3*, *red1*	Gerald Fink lab
yCW816	*MAT* **aa** isogenic to L5486	This study
L5539	L5486 with *kss1Δ*::*LEU2*, *fus3Δ*::*LEU2*, *HIS3*, cured of plasmid	Gerald Fink lab
yCW809	*MAT* **aa** isogenic to L5539 without plasmid	This study
PY3295	*MAT* **a**, identical to BY4741 (WT S288c), *his3Δ*, *leu2Δ*, *met15Δ*, *ura3Δ*, *LYS2*	David Pellman lab
PY5006	*MAT* **aaaa**, isogenic to PY3295 except *MET15*/*MET15*/*met15Δ*/*met15Δ*, *LYS2*/*LYS2*/*lys2Δ*/*lys2*Δ	David Pellman lab
BYC0037	*MAT* **a**, BY4741 (S288c) with an integrated functional allele of *FLO8*	Gerald Fink lab
yCW834	*MAT* **aaaa**, isogenic to BYC0037	This study

All strains are in the Σ1278b background with the genotype *ura3-52*, *leu2*::*hisG*, *his3*::*hisG* unless noted otherwise.

### Preparation of cDNA Libraries for Sequencing

Total RNA was extracted from yeast cultures in mid-log phase with acidic phenol. After enrichment of poly(A) RNA (Qiagen Oligotex mRNA kit), the resultant mRNA was processed for cDNA library construction and sequencing as previously described [11]. The libraries were sequenced for 36 cycles on Illumina Genome Analyzer 2 using the standard protocol.

### Mapping Algorithm for RNA-Seq Reads

Reads were mapped to the Σ1278b genome using the Bowtie alignment software (version 0.10.0; http://bowtie-bio.sourceforge.net/index.shtml). Reads were either mapped uniquely (bowtie –solexa-quals -k 1 -m 1 –best –strata -p 2 –strandfix) or multiply (bowtie –solexa-quals -k 100 -m 100 –best –strata -p 2 –strandfix). We used unique mappings only to look at differential gene expression. Multiple mappings were used to assess how many reads aligned to TY elements, the rDNA cluster, and other repetitive sequences; a read that mapped to *n* genomic locations was assigned a weight of 1/*n*, and the “number of reads” mapping to a repetitive element was the sum of the weights of the hits in that element. The complete RNA-seq data are available at the Gene Expression Omnibus (http://www.ncbi.nlm.nih.gov/geo/) repository with the accession number GSE19685.

### Enriching Differentially Expressed Candidate Genes in the RNA-Seq Data

Mapped reads were stored in the David K. Gifford group's in-house ChIP/RNA-seq database and analyzed with the code provided in DifferentialExpression.java. This code performs the following procedure on each annotated ORF in the Σ1278b genome. (1) Determine the total number of uniquely mapped reads in the haploid and tetraploid experiments. (2) Determine the number of uniquely mapped reads on both strands in the ORF in the haploid and tetraploid experiments. (3) Compute frequency_haploid = (haploid count for gene)/(total haploid count) and frequency_tetraploid = (tetraploid count for gene)/(total tetraploid count). (4) Use frequency_haploid to compute a *p*-value for the observed reads in the tetraploid experiment using a binomial model given the frequency in the haploid experiment. That is
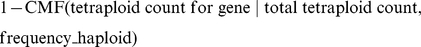
The CMF is the cumulative mass function (the discrete equivalent of a cumulative distribution function) and is the sum of the probabilities for all counts less than or equal to the observed count. This is the *p*-value for the haploid observation given the tetraploid observation. (5) Compute the *p*-value for the tetraploid observation given the haploid observation. (6) Retain genes with *p*<0.001.

The worksheets “sample pair A” and “sample pair B” in Dataset S1 show the results.

### Identification of Genes Transcriptionally Affected by Ploidy in the RNA-Seq Data

Based on read counts of known silenced genes (hypoxia response and sporulation specific), a threshold of 15 was set as the minimal expressed level. In total, 5,613 genes were considered expressed and constituted the “background gene list” for subsequent GO analysis on *Saccharomyces* Genome Database (SGD). The list of differentially expressed genes with read count of 15 or greater provided the set of candidate genes.

The differentially regulated candidate genes were sorted by fold change after gene read counts had been normalized by the total number of reads in each dataset. Equal numbers of the top-ranking candidates from the two haploid–tetraploid replicates were compared, and the overlapping candidates were identified as differentially regulated. The numbers of top-ranking candidates from the replicates were selected to obtain a sufficient number of overlapping genes for GO analysis while ensuring that the overlap between replicates was highly statistically significant (*p*<e^−10^) by hypergeomtric test using MATLAB (MathWorks).

### Binding Motif Analysis for Genes Disproportionally Expressed in the Tetraploid

Given the list of differentially expressed genes, we scanned the upstream promoter region for all motifs published [41]. For each gene, we determined the promoter region as the region (1) extending 50 bp downstream of the annotated coding start site and (2) extending upstream of the annotated coding start site to the first annotated feature (ORF, Ty, tRNA, etc.) or 5 kb away. Features that overlap the transcription start site are not used as the first upstream feature.

For each motif, we determined the numerical score of the log-likelihood matrix match against all genes as the best match of the motif to the upstream promoter. Each log-likelihood matrix has a positive maximum score representing the highest possible value that the matrix can assign to a DNA sequence seen while scanning; as these are log-likelihood matrices, a score of zero indicates that the sequence in question matches the background model as well as it matches the matrix. The motif-scanning code then tested cutoff scores between 0.3 and 1.0 (in increments of 0.05) times the maximum possible score for the motif to determine the score to use to call a motif “match”; the score cutoff used was the one that produced the most significant result when comparing the motif frequency in genes up-regulated in tetraploid against motif frequency in all genes using a binomial test. We performed a similar process on the down-regulated genes.

We filtered the results to include only motifs that showed a *p*-value of 0.005 or less, were found in at least 25% of up-/down-regulated genes, and for which the fold change in frequency was at least 1.5. We used these filters to retain motifs mostly likely to be relevant to the lists of identified genes.

Dataset S2 shows the results of the motif scanning.

### Quantifying Expression Levels of Specific Genes by PCR

Total RNA extracted using acidic phenol was processed for cDNA synthesis using the QuantiTect Reverse Transcription Kit (Qiagen). Expression levels were measured on an Applied Biosystems 7500 Real-Time PCR System with SYBR Green in Absolute Quantification mode following manufacture's procedure. Unless specified, we used the abundance of *ACT1* transcript to normalize expression levels of genes of interest. The representation of *ACT1* transcript in total RNA is constant in all strains used in this study. Statistical treatment (unpaired *t* test for two-tailed *p*-values) of qPCR data was performed using GraphPad Prism (GraphPad Software).

### Measurement of Cell Size

Cells were fixed in 3.7% formaldehyde at 4°C overnight and digested with a mixture of zymolyase and glusulase in the presence of 1.2 M sorbitol citrate to relieve aggregation. Microscopy images of more than 50 cells per strain were analyzed using ImageJ (United States National Institutes of Health). In experiments involving cell cycle arrest with nocodazole, cell size was calculated from the measured width and length of both mother and bud of large budded cells, assuming rotational symmetry about the long axis. For actively cycling cultures, cell size was calculated from the measured width and length of the mother of budded cells. Statistical comparison of cell size was performed using Student's *t* test.

### Construction of Isogenic *MAT*aa Diploids

Haploid strains with suitable genotypes were transformed with a plasmid encoding the HO endonuclease inducible by galatose to enable mating-type switching. Transformed strains were pre-grown overnight in synthetic complete drop-out medium supplemented with 0.1% glucose. After sufficient washing with water, cells were resuspended in synthetic complete drop-out medium plus 2% galactose and grown for approximately one doubling time to enable mating-type switching before plating on YPD agar. Mating-type-switched candidates were passaged multiple times on YPD to ensure loss of the HO-encoding plasmid prior to mating-type assessment by auxotrophic marker complementation or a pheromone-dependent growth inhibition assay.

## Supporting Information

Dataset S1 **Results for the RNA-seq comparison between the transcriptomes of haploid and tetraploid cells.** In the worksheets “sample pair A” and “sample pair B,” read counts of annotated ORFs in the two pairs of RNA-seq datasets are listed. Also included are *p*-values for calling differentially expressed genes and the sample total read counts. The worksheet “gene lists” shows genes in the “background list” for GO analysis and the lists of genes differentially regulated in tetraploids sorted by average fold change in the RNA-seq datasets. The remaining worksheets show the complete GO term search results from SGD for genes differentially expressed in tetraploids. GO terms for biological process, molecular function, and cellular compartment are listed separately.
(2.33 MB XLS)

Dataset S2 **Overrepresented transcription factor motifs in the promoters of differentially regulated genes.** Worksheet “Dig1, Ste12, Mcm1” shows the likelihood of these transcription factors binding to the promoters of interest. The remaining worksheets show motifs found to be significantly enriched in specific gene sets.
(0.05 MB XLS)

Table S1 **Regulation of disproportionally expressed genes in the tetraploid is not correlated with stages in the mitotic cell cycle.**
(0.07 MB DOC)

Table S2 **Cellular compartment GO terms for genes repressed in the tetraploid.**
(0.03 MB DOC)

Table S3 **Biological process GO terms for genes repressed in the tetraploid.**
(0.03 MB DOC)

Table S4 **Molecular function GO terms for genes repressed in the tetraploid.**
(0.03 MB DOC)

Table S5 **GO terms for genes induced in the tetraploid.**
(0.03 MB DOC)

Table S6 **Mitotic arrest efficiency, measured as percentages of arrested cells, in experiments shown in** **Figure 3C–3E** **.**
(0.03 MB DOC)

Table S7 **Expression levels of genes down-regulated in the WT tetraploid in the** ***cln3*** **Δ haploid.**
(0.06 MB DOC)

Table S8 **Expression levels of genes up-regulated in the WT tetraploid in the** ***cln3*** **Δ haploid.**
(0.06 MB DOC)

Table S9 **Relationship between cell size/surface area and gene expression analyzed with linear regression.**
(0.05 MB DOC)

Table S10 **The binding motifs of Dig1 and Ste12 are overrepresented in the promoters of genes repressed in the Σ1278b tetraploid.**
(0.05 MB DOC)

Text S1 **Cell size mutants incompatible with cell size–transcription analysis.**
(0.03 MB DOC)

Text S2 **Discussion on genes up-regulated in large cells.**
(1.08 MB DOC)

## Abbreviations

GO: Gene Ontology
MAPK: mitogen-activated protein kinase
qPCR: quantitative PCR
SGD: *Saccharomyces* Genome Database
WT: wild type
YPD: yeast extract peptone dextrose
