# Supplementary material for: Control of Transcription by Cell Size
Source: PLoS Biol. 2010 Nov 2;8(11):e1000523. doi: 10.1371/journal.pbio.1000523 (PMC2970550; doi:10.1371/journal.pbio.1000523)
Supplement: Table S1 — Regulation of disproportionally expressed genes in the tetraploid is not correlated with stages in the mitotic cell cycle. (0.07 MB DOC) [file pbio.1000523.s003.doc]

**Supporting Table 1.** Regulation of disproportionally expressed genes in the tetraploid is not correlated with stages in the mitotic cell cycle. Except for *FLO11*, cell cycle regulation information was obtained from a previous genome wide study [43].

**Part A**. Genes repressed in the tetraploid and the cell cycle stages at which their expression levels are maximal.

* denotes genes whose fluctuation in transcript abundance did not exceed a defined threshold to be considered cell cycle regulated in the study.

| ORF | Symbol | Peak expression |
| --- | --- | --- |
| *YIR019C* | *FLO11* | M |
| *YLR042C* |  | (no effect) |
| *YOL154W* | *ZPS1* | * |
| *YDR461W* | *MFA1* | G1 |
| *YNR060W* | *FRE4* | * |
| *YFL026W* | *STE2* | M |
| *YCL027W* | *FUS1* | M/G1 |
| *YGL193C* |  | * |
| *YBL016W* | *FUS3* | * |
| *YER150W* | *SPI1* | M/G1 |
| *YGL032C* | *AGA2* | M/G1 |
| *YIL015W* | *BAR1* | * |
| *YLR040C* |  | M/G1 |
| *YMR173W* | *DDR48* | * |
| *YKR013W* | *PRY2* | G1 |
| *YKL209C* | *STE6* | M |
| *YLR452C* | *SST2* | M/G1 |
| *YNL160W* | *YGP1* | M/G1 |
| *YNR044W* | *AGA1* | M/G1 |
| *YPL163C* | *SVS1* | G1 |
| *YNL145W* | *MFA2* | M |
| *YOL104C* | *NDJ1* | * |
| *YOR212W* | *STE4* | * |
| *YHL043W* | *ECM34* | * |
| *YDL227C* | *HO* | G1 |
| *YKL096W-A* | *CWP2* | G2 |
| *YFR022W* | *ROG3* | * |
| *YHR005C* | *GPA1* | M/G1 |
| *YBR054W* | *YRO2* | M |
| *YDR309C* | *GIC2* | G1 |
| *YMR266W* | *RSN1* | * |
| *YFL027C* | *GYP8* | * |
| *YGR014W* | *MSB2* | G1 |
| *YMR305C* | *SWC10* | G1 |
| *YIL140W* | *AXL2* | G1 |

Peak expression Number of genes

G1 8

G2 1

M 5

M/G1 8

**Part B.** Genes disproportionally induced in the tetraploid and the cell cycle stages at which their expression levels are maximal.

| ORF | Symbol | Peak expression |
| --- | --- | --- |
| *YER124C* | *DSE1* | G1 |
| *YHR143W* | *DSE2* | G1 |
| *YLR286C* | *CTS1* | G1 |
| *YGL028C* | *SCW11* | G1 |
| *YJR109C* | *CPA2* | * |
| *YIR039C* | *YPS6* | * |
| *YIL169C* | *YIL169C* | * |
| *YNR067C* | *DSE4* | M/G1 |
| *YIR009W* | *MSL1* | * |
| *YLR285C-A* |  | no data |
| *YDR360W* | *OPI7* | (no effect) |
| *YKR076W* | *ECM4* | * |
| *YCL026C-B* | *HBN1* | no data |
| *YNL122C* |  | * |
| *YDR379C-A* |  | no data |
| *YBR120C* | *CBP6* | * |
| *YJL023C* | *PET130* | * |
| *YBL059C-A* | *CMC2* | no data |
| *YIL098C* | *FMC1* | * |
| *YOR195W* | *SLK19* | G1 |
| *YJL200C* | *ACO2* | * |
| *YER068C-A* |  | no data |
| *YCR003W* | *MRPL32* | * |
| *YDL044C* | *MTF2* | * |
| *YOR216C* | *RUD3* | * |
| *YLR254C* | *NDL1* | G2/M |
| *YDR357C* |  | * |
| *YMR180C* | *CTL1* | * |
| *YCL056C* | *YCL056C* | * |
| *YCR096C* | *A2* | * |

* denotes genes whose fluctuation in transcript abundance did not exceed a defined threshold to be considered cell cycle regulated in the study.

Peak expression Number of genes

G1 5

G2/M 2

M/G1 1
