# Supplementary material for: Control of Transcription by Cell Size
Source: PLoS Biol. 2010 Nov 2;8(11):e1000523. doi: 10.1371/journal.pbio.1000523 (PMC2970550; doi:10.1371/journal.pbio.1000523)
Supplement: Table S2 — Cellular compartment GO terms for genes repressed in the tetraploid. (0.03 MB DOC) [file pbio.1000523.s004.doc]

**Supporting Table 2.** Cellular compartmental GO terms for genes repressed in the tetraploid.

| GO term | Cluster frequency | Background frequency | p-value | Genes |
| --- | --- | --- | --- | --- |
| Cell wall | 10/35, 28.6% | 80/5613, 1.4% | 2.7 e-11 | *AGA1, AGA2,*  *ZPS1, SPI1,*  *SVS1, YGP1,*  *CWP2, SCW10,*  *YLR040C,*  *FLO11* |
| Plasma  membrane | 11/35, 31.4% | 251/5613, 4.5% | 1.9 e-7 | *FRE4,*  *STE2, FUS1,*  *STE6, SST2,*  *STE4, GPA1,*  *GIC2, MSB2,*  *AXL2, RSN1* |
