# Supplementary material for: Control of Transcription by Cell Size
Source: PLoS Biol. 2010 Nov 2;8(11):e1000523. doi: 10.1371/journal.pbio.1000523 (PMC2970550; doi:10.1371/journal.pbio.1000523)
Supplement: Table S3 — Biological process GO terms for genes repressed in the tetraploid. (0.03 MB DOC) [file pbio.1000523.s005.doc]

**Supporting Table 3.** GO terms in biological processes for genes repressed in the tetraploid.

| GO term | Cluster frequency | Background frequency | p-value | Genes |
| --- | --- | --- | --- | --- |
| Mating & adhesion | 13/35, 37.1% | 123/5613, 2.2% | 1.4 e-13 | *FLO11, MFA1,*  *MFA2, STE2,*  *STE4, FUS1,*  *FUS3, AGA1,*  *AGA2, BAR1,*  *SST2, GPA1,*  *SCW10* |
| Cell surface  receptor linked  signal transduction | 7/35, 20% | 50/5613, 0.9% | 1.6 e-8 | *MFA1, MFA2,*  *STE2, STE4,*  *FUS3, GPA1,*  *MSB2* |
