# Supplementary material for: Control of Transcription by Cell Size
Source: PLoS Biol. 2010 Nov 2;8(11):e1000523. doi: 10.1371/journal.pbio.1000523 (PMC2970550; doi:10.1371/journal.pbio.1000523)
Supplement: Table S4 — Molecular function GO terms for genes repressed in the tetraploid. (0.03 MB DOC) [file pbio.1000523.s006.doc]

**Supporting Table 4.** GO terms in molecular functions for genes repressed in the tetraploid.

| GO term | Cluster frequency | Background frequency | p-value | Genes |
| --- | --- | --- | --- | --- |
| Receptor binding | 2/35, 5.7% | 2/5613, 0.03% | 3.8 e-5 | *MFA1, MFA2* |
| Cell adhesion molecule binding | 2/35, 5.7% | 3/5613, 0.1% | 1.1 e-4 | *AGA1, AGA2* |
| Signal transducer | 4/35, 11.4% | 42/5613, 0.7% | 1.2 e-4 | *FUS3, MSB2,*  *STE2, STE4* |
