# Supplementary material for: Control of Transcription by Cell Size
Source: PLoS Biol. 2010 Nov 2;8(11):e1000523. doi: 10.1371/journal.pbio.1000523 (PMC2970550; doi:10.1371/journal.pbio.1000523)
Supplement: Table S5 — GO terms for genes induced in the tetraploid. (0.03 MB DOC) [file pbio.1000523.s007.doc]

**Supporting Table 5.** GO terms for genes induced in the tetraploid.

| GO term | Cluster frequency | Background frequency | p-value | Genes |
| --- | --- | --- | --- | --- |
| Cytokinesis  (process) | 5/30, 16.7% | 11/5613, 0.2% | 1.4 e-9 | *DSE1,DSE2,*  *DSE4, CTS1,*  *SCW11* |
| Hydrolase  (function) | 4/30, 13.3% | 30/5613, 0.5% | 1.7 e-5 | *DSE2, DSE4,*  *CTS1,SCW11* |
| Cell wall  (compartment) | 5/30, 16.7% | 80/5613, 1.4% | 5.6 e-5 | *DSE2, DSE4,*  *CTS1,SCW11,*  *YPS6* |
