# Supplementary material for: Control of Transcription by Cell Size
Source: PLoS Biol. 2010 Nov 2;8(11):e1000523. doi: 10.1371/journal.pbio.1000523 (PMC2970550; doi:10.1371/journal.pbio.1000523)
Supplement: Table S6 — Mitotic arrest efficiency, measured as percentages of arrested cells, in experiments shown in Figure 3C–3E . (0.03 MB DOC) [file pbio.1000523.s008.doc]

**Supporting Table 6.** Mitotic arrest efficiency, measured as percentages of arrested cells, in experiments shown in figures 3C-E. For each strain, at least 250 cells were counted from each of the three biological replicate cultures. Averages and standard deviations of the percentages were then calculated.

For figure 3C:

|  | % unbudded | % small budded | % large budded |
| --- | --- | --- | --- |
| WT | 15.48 ± 2.29 | 4.35 ± 0.68 | 80.18 ± 2.40 |
| *bck2*∆ | 17.99 ± 1.08 | 2.94 ± 0.73 | 79.08 ± 1.71 |

For figure 3D:

|  | % unbudded | % small budded | % large budded |
| --- | --- | --- | --- |
| WT | 19.26 ± 2.33 | 5.62 ± 1.01 | 75.12 ± 3.27 |
| *eap1*∆ | 11.07 ± 2.68 | 5.59 ± 1.74 | 83.34 ± 1.21 |

For figure 3E:

|  | % unbudded | % small budded | % large budded |
| --- | --- | --- | --- |
| *CLN3-2* | 17.99 ± 2.31 | 4.82 ± 1.54 | 77.19 ± 0.80 |
| WT | 17.73 ± 4.65 | 6.64 ± 1.56 | 75.63 ± 3.27 |
| *cln3*∆ | 18.82 ± 3.21 | 6.02 ± 1.49 | 75.16 ± 2.37 |
