# Supplementary material for: Control of Transcription by Cell Size
Source: PLoS Biol. 2010 Nov 2;8(11):e1000523. doi: 10.1371/journal.pbio.1000523 (PMC2970550; doi:10.1371/journal.pbio.1000523)
Supplement: Table S7 — Expression levels of genes down-regulated in the WT tetraploid in the cln3 Δ haploid. (0.06 MB DOC) [file pbio.1000523.s009.doc]

**Supporting Table 7.** Regulation of genes repressed in the WT tetraploid in the *cln3*∆ haploid.

**Part A.** The majority of genes identified in figure 2A show the same regulatory trend in the WT tetraploid and the *cln3*∆ haploid.

| Regulation | Gene | 1n Ave ± SD | 4n Ave ± SD | Sig. | WT Ave ± SD | *cln3*∆ Ave ± SD | Sig. |
| --- | --- | --- | --- | --- | --- | --- | --- |
| $ | *FLO11* | 10.196 ± 1.007 | 1 ± 0.325 | *** | 4.925 ± 0.490 | 1 ± 0.169 | *** |
| $ | *YLR042C* | 9.994 ± 0.954 | 1 ± 0.140 | *** | 3.826 ± 0.601 | 1 ± 0.119 | ** |
| $ | *MFA1* | 3.481 ± 0.122 | 1 ± 0.113 | *** | 2.768 ± 0.169 | 1 ± 0.080 | *** |
| $ | *FRE4* | 13.211 ± 1.531 | 1 ± 0.806 | *** | 7.171 ± 1.772 | 1 ± 0.670 | * |
| $ | *STE2* | 2.922 ± 0.106 | 1 ± 0.063 | *** | 1.870 ± 0.100 | 1 ± 0.055 | *** |
| $ | *FUS1* | 3.199 ± 0.189 | 1 ± 0.269 | *** | 2.190 ± 0.242 | 1 ± 0.015 | *** |
| $ | *FUS3* | 4.302 ± 0.344 | 1 ± 0.143 | *** | 2.416 ± 0.167 | 1 ± 0.070 | *** |
| $ | *AGA2* | 2.987 ± 0.171 | 1 ± 0.207 | *** | 2.059 ± 0.069 | 1 ± 0.099 | *** |
| $ | *BAR1* | 2.025 ± 0.200 | 1 ± 0.103 | ** | 1.982 ± 0.207 | 1 ± 0.244 | ** |
| $ | *YLR040C* | 2.808 ± 0.199 | 1 ± 0.115 | *** | 2.494 ± 0.226 | 1 ± 0.034 | *** |
| $ | *DDR48* | 3.418 ± 0.196 | 1 ± 0.161 | *** | 1.875 ± 0.104 | 1 ± 0.071 | *** |
| $ | *RPY2* | 2.354 ± 0.334 | 1 ± 0.023 | ** | 1.788 ± 0.059 | 1 ± 0.046 | *** |
| $ | *STE6* | 2.119 ± 0.144 | 1 ± 0.068 | *** | 1.636 ± 0.191 | 1 ± 0.092 | ** |
| $ | *SST2* | 2.230 ± 0.063 | 1 ± 0.079 | *** | 2.331 ± 0.080 | 1 ± 0.037 | *** |
|  | *AGA1* | 2.157 ± 0.389 | 1 ± 0.077 | ** | 1.360 ± 0.208 | 1 ± 0.119 |  |
| $ | *SVS1* | 2.490 ± 0.172 | 1 ± 0.048 | *** | 2.219 ± 0.291 | 1 ± 0.070 | ** |
| $ | *MFA2* | 1.638 ± 0.251 | 1 ± 0.059 | * | 1.467 ± 0.012 | 1 ± 0.031 | *** |
| $ | *NDJ1* | 1.492 ± 0.114 | 1 ± 0.046 | ** | 1.610 ± 0.282 | 1 ± 0.238 | * |
| $ | *STE4* | 1.808 ± 0.101 | 1 ± 0.109 | *** | 1.776 ± 0.179 | 1 ± 0.235 | * |
|  | *HO* | 2.655 ± 0.176 | 1 ± 0.082 | *** | 0.895 ± 0.131 | 1 ± 0.049 |  |
| $ | *CWP2* | 1.658 ± 0.105 | 1 ± 0.031 | *** | 1.594 ± 0.054 | 1 ± 0.063 | *** |
|  | *GPA1* | 1.861 ± 0.145 | 1 ± 0.059 | *** | 1.275 ± 0.131 | 1 ± 0.161 |  |
| $ | *GIC2* | 1.680 ± 0.046 | 1 ± 0.047 | *** | 1.793 ± 0.187 | 1 ± 0.046 | ** |
| $ | *RSN1* | 1.584 ± 0.059 | 1 ± 0.091 | *** | 1.748 ± 0.085 | 1 ± 0.036 | *** |
|  | *GYP8* | 2.014 ± 0.115 | 1 ± 0.101 | *** | 1.241 ± 0.193 | 1 ± 0.012 |  |
|  | *MSB2* | 1.370 ± 0.124 | 1 ± 0.016 | ** | 2.722 ± 0.873 | 1 ± 0.760 |  |
|  | *SCW10* | 1.515 ± 0.067 | 1 ± 0.023 | *** | 0.987 ± 0.044 | 1 ± 0.026 |  |

Transcript expression levels were measured from total RNA by quantitative PCR. WT haploid and tetraploid were cultured asynchronously in YPD, whereas WT and *cln3*∆ haploids were cultured in YPD + nocodazole. Listed in the table are genes that remained expressed and down-regulated in the tetraploid in the YPD medium, among those identified in figure 2A. Gene expression levels in WT haploids were normalized to those in the tetraploid or the *cln3*∆ haploid. Hence, the average expression level of a gene in the WT haploid represents the average fold change in expression. Significance of differences in expression levels were analyzed by Student’s t-test (n=3). Statistical annotations used in the “Sig.” columns: *** for p-value less than 0.001. ** for p-value between 0.01 and 0.001. * for p-value between 0.05 and 0.01. In the “Regulation” column, $ denotes genes regulated in the same trend in the WT tetraploid and the *cln3*∆ haploid as compared with the WT haploid.

**Part B.** The difference in *FLO11* expression levels between the *cln3*∆ haploid and the WT tetraploid shown in Part A correlates with cell size, not differential treatment with nocodazole.

| Strain | expression levels (Ave ± SD) | % large budded cells | % unbud + small bud |
| --- | --- | --- | --- |
| WT haploid | 1.00 ± 0.170 | 73.3 | 26.7 |
| *cln3*∆ haploid | 0.16 ± 0.016 | 72.5 | 27.5 |
| WT tetraploid | 0.08 ± 0.012 | 65.4 | 34.6 |

The *MAT***a** WT haploid, *cln3*∆ haploid and WT tetraploid were cultured in the presence of nocodazole and processed as described in figure 3. The fold changes in *cln3*∆ haploid and in WT tetraploid in the presence of nocodazole were comparable to those shown in Part A. As tetraploid cells arrested less efficiently and had a noticeably higher % of cells in G1 and S under the experimental condition, transcripts maximally expressed in those cell cycle stages could be artifactually over-represented in the tetraploid samples. Due to this concern and the fact that nocodazole did not significantly alter the fold changes in *FLO11* expression, the asynchronous growth condition was employed for the haploid vs tetraploid analysis.
