# Supplementary material for: Control of Transcription by Cell Size
Source: PLoS Biol. 2010 Nov 2;8(11):e1000523. doi: 10.1371/journal.pbio.1000523 (PMC2970550; doi:10.1371/journal.pbio.1000523)
Supplement: Table S8 — Expression levels of genes up-regulated in the WT tetraploid in the cln3 Δ haploid. (0.06 MB DOC) [file pbio.1000523.s010.doc]

**Supporting Table 8. Expression levels of genes up-regulated in the WT tetraploid in the *cln3*∆ haploid.**

| Regulation | Gene | 1n Ave ± SD | 4n Ave ± SD | Sig. | WT Ave ± SD | *cln3*∆ Ave ± SD | Sig. |
| --- | --- | --- | --- | --- | --- | --- | --- |
| $ | *DSE1* | 1 ± 0.025 | 19.661 ± 4.019 | ** | 1 ± 0.120 | 5.503 ± 0.133 | *** |
| $ | *DSE2* | 1 ± 0.075 | 14.086 ± 0.450 | *** | 1 ± 0.131 | 2.585 ± 0.048 | *** |
| $ | *CTS1* | 1 ± 0.097 | 12.793 ± 0.775 | *** | 1 ± 0.319 | 2.770 ± 0.894 | * |
| $ | *SCW11* | 1 ± 0.063 | 6.317 ± 0.605 | *** | 1 ± 0.022 | 2.085 ± 0.089 | *** |
| $ | *CPA2* | 1 ± 0.132 | 2.205 ± 0.140 | *** | 1 ± 0.137 | 2.020 ± 0.142 | *** |
| $ | *YPS6* | 1 ± 0.030 | 5.165 ± 0.630 | *** | 1 ± 0.095 | 4.157 ± 0.330 | *** |
| $ | *YIL169C* | 1 ± 0.078 | 4.989 ± 0.289 | *** | 1 ± 0.282 | 3.640 ± 0.575 | ** |
|  | *DSE4* | 1 ± 0.021 | 3.375 ± 0.277 | *** | 1 ± 0.135 | 1.453 ± 0.409 |  |
|  | *MSL1* | 1 ± 0.341 | 5.194 ± 1.688 | * | 1 ± 0.068 | 2.004 ± 0.909 |  |
|  | *OPI7* | 1 ± 0.522 | 3.064 ± 0.607 | * | 1 ± 0.239 | 0.637 ± 0.137 |  |
| $ | *ECM4* | 1 ± 0.028 | 2.511 ± 0.294 | *** | 1 ± 0.156 | 2.270 ± 0.055 | *** |
| $ | *HBN1* | 1 ± 0.040 | 3.108 ± 0.264 | *** | 1 ± 0.175 | 2.007 ± 0.031 | *** |
|  | *YNL122C* | 1 ± 0.074 | 1.527 ± 0.094 | ** | 1 ± 0.164 | 0.874 ± 0.027 |  |
|  | *YDR379C-A* | 1 ± 0.049 | 3.136 ± 0.063 | *** | 1 ± 0.105 | 1.207 ± 0.220 |  |
|  | *CBP6* | 1 ± 0.500 | 3.747 ± 0.640 | ** | 1 ± 0.148 | 0.960 ± 0.045 |  |
| ^ | *CMC2* | 1 ± 0.164 | 1.755 ± 0.029 | ** | 1 ± 0.114 | 0.703 ± 0.051 | * |
|  | *FMC1* | 1 ± 0.030 | 1.513 ± 0.213 | * | 1 ± 0.147 | 0.855 ± 0.037 |  |
|  | *SLK19* | 1 ± 0.039 | 1.365 ± 0.101 | ** | 1 ± 0.273 | 0.992 ± 0.112 |  |
|  | *ACO2* | 1 ± 0.084 | 2.767 ± 0.312 | *** | 1 ± 0.209 | 0.654 ± 0.085 |  |
|  | *MRPL32* | 1 ± 0.703 | 4.803 ± 0.782 | ** | 1 ± 0.163 | 0.782 ± 0.118 |  |
|  | *MTF2* | 1 ± 0.071 | 1.227 ± 0.094 | * | 1 ± 0.175 | 0.816 ± 0.038 |  |
|  | *RUD3* | 1 ± 0.056 | 1.155 ± 0.038 | * | 1 ± 0.257 | 0.824 ± 0.088 |  |
|  | *NDL1* | 1 ± 0.165 | 1.321 ± 0.075 | * | 1 ± 0.247 | 0.868 ± 0.021 |  |
|  | *YDR357C* | 1 ± 0.023 | 1.394 ± 0.106 | ** | 1 ± 0.210 | 1.085 ± 0.017 |  |
|  | *CTL1* | 1 ± 0.226 | 1.670 ± 0.107 | ** | 1 ± 0.163 | 1.032 ± 0.038 |  |

Transcript expression levels were measured from total RNA by quantitative PCR. WT haploid and tetraploid were cultured asynchronously in YPD, whereas WT and *cln3*∆ haploids were cultured in YPD + nocodazole. Listed in the table are genes that remained expressed and up-regulated in the tetraploid in YPD, among those identified in figure 2B. Gene expression levels in the tetraploid and the *cln3*∆ haploid were normalized to those in the WT haploid. The average expression level of a gene in the tetraploid or the *cln3*∆ haploid thus represents the average fold change in expression. Significance of differences in expression levels were analyzed by Student’s t-test (n=3). Statistical annotations used in the “Sig.” columns: *** for p-value less than 0.001. ** for p-value between 0.01 and 0.001. * for p-value between 0.05 and 0.01. In the “Regulation” column, $ denotes genes regulated in the same trend in the WT tetraploid and the *cln3*∆ haploid as compared with the WT haploid. ^ denotes genes regulated in the opposite trend.
