# Supplementary material for: Control of Transcription by Cell Size
Source: PLoS Biol. 2010 Nov 2;8(11):e1000523. doi: 10.1371/journal.pbio.1000523 (PMC2970550; doi:10.1371/journal.pbio.1000523)
Supplement: Table S9 — Relationship between cell size/surface area and gene expression analyzed with linear regression. (0.05 MB DOC) [file pbio.1000523.s011.doc]

**Supporting Table 9.** Relationship between cell size/surface area and gene expression analyzed with linear regression.

Linear regression was performed using the relative cell size or cell surface area as X values. Relative cell size: WT haploid: 1, *bck2*∆: 1.24, *eap1*∆: 1.37, *cln3*∆: 1.85, WT tetraploid: 4. Relative cell surface area, based on cell size and the assumption that cells are spherical: WT haploid: 1, *bck2*∆: 1.14, *eap1*∆: 1.23, *cln3*∆: 1.51, WT tetraploid: 2.53.

The Y values were the relative gene expression levels as shown in figures 4 and 5.

We did not model the relationship between gene expression and cell size using higher order polynomials because there are not enough of cell size variants to generate a sufficient number of data points for proper curve fitting.

|  | X = relative cell size | | | X = relative surface area | | |
| --- | --- | --- | --- | --- | --- | --- |
| **Gene** | **r^2** | **Slope** | **Y-intercept** | **r^2** | **Slope** | **Y-intercept** |
| *FLO11* | 0.272 | -0.17 ± 0.16 | 0.67 ± 0.35 | 0.304 | -0.35 ± 0.31 | 0.88 ± 0.49 |
| *YLR042C* | 0.489 | -0.33 ± 0.20 | 1.35 ± 0.43 | 0.502 | -0.67 ± 0.38 | 1.71 ± 0.61 |
| *MFA1* | 0.656 | -0.20 ± 0.08 | 1.01 ± 0.18 | 0.704 | -0.41 ± 0.15 | 1.23 ± 0.24 |
| *FRE4* | 0.255 | -0.16 ± 0.16 | 0.62 ± 0.34 | 0.284 | -0.33 ± 0.31 | 0.81 ± 0.48 |
| *STE2* | 0.799 | -0.18 ± 0.05 | 1.03 ± 0.12 | 0.835 | -0.37 ± 0.10 | 1.24 ± 0.15 |
| *FUS1* | 0.726 | -0.19 ± 0.07 | 1.00 ± 0.15 | 0.768 | -0.38 ± 0.12 | 1.22 ± 0.19 |
| *FUS3* | 0.771 | -0.23 ± 0.07 | 1.10 ± 0.16 | 0.805 | -0.47 ± 0.13 | 1.36 ± 0.21 |
| **Gene** | **r^2** | **Slope** | **Y-intercept** | **r^2** | **Slope** | **Y-intercept** |
| *DSE1* | 0.914 | 5.53 ± 0.98 | -2.64 ± 2.14 | 0.909 | 10.94 ± 2.00 | -8.38 ± 3.16 |
| *DSE2* | 0.977 | 4.58 ± 0.40 | -4.61 ± 0.88 | 0.960 | 8.99 ± 1.06 | -9.27 ± 1.68 |
| *CTS1* | 0.985 | 4.08 ± 0.30 | -3.81 ± 0.64 | 0.970 | 8.03 ± 0.82 | -7.98 ± 1.29 |
| *SCW11* | 0.975 | 1.80 ± 0.17 | -0.94 ± 0.36 | 0.969 | 3.56 ± 0.37 | -2.81 ± 0.58 |
| *CPA2* | 0.688 | 0.40 ± 0.16 | 0.73 ± 0.34 | 0.730 | 0.82 ± 0.29 | 0.27 ± 0.46 |
| *YPS6* | 0.760 | 1.42 ± 0.46 | -0.15 ± 1.01 | 0.790 | 2.87 ± 0.85 | -1.72 ± 1.35 |
| *YIL169C* | 0.814 | 1.42 ± 0.39 | -0.39 ± 0.85 | 0.840 | 2.85 ± 0.72 | -1.94 ± 1.14 |
