# Supplementary material for: Control of Transcription by Cell Size
Source: PLoS Biol. 2010 Nov 2;8(11):e1000523. doi: 10.1371/journal.pbio.1000523 (PMC2970550; doi:10.1371/journal.pbio.1000523)
Supplement: Table S10 — The binding motifs of Dig1 and Ste12 are overrepresented in the promoters of genes repressed in the Σ1278b tetraploid. (0.05 MB DOC) [file pbio.1000523.s012.doc]

**Supporting Table 10.** The binding motifs of Dig1 and Ste12 are over-represented in the promoters of genes repressed in the Σ1278b tetraploid.

| Gene | Dig1 motif | Ste12 motif |
| --- | --- | --- |
| *FLO11* | NO | YES |
| *YLR042C* | YES | YES |
| *ZPS1* | NO | YES |
| *MFA1* | YES | YES |
| *FRE4* | NO | NO |
| *STE2* | NO | YES |
| *FUS1* | YES | YES |
| *YGL193C* | NO | NO |
| *FUS3* | YES | YES |
| *SPI1* | NO | YES |
| *AGA2* | NO | YES |
| *BAR1* | YES | YES |
| *YLR040C* | NO | NO |
| *DDR48* | NO | NO |
| *PRY2* | YES | YES |
| *STE6* | YES | YES |
| *SST2* | YES | YES |
| *YGP1* | NO | YES |
| *AGA1* | YES | YES |
| *SVS1* | NO | YES |
| *MFA2* | YES | YES |
| *NDJ1* | NO | NO |
| *STE4* | YES | YES |
| *ECM34* | NO | NO |
| *HO* | NO | YES |
| *CWP2* | NO | NO |
| *ROG3* | NO | NO |
| *GPA1* | NO | YES |
| *YRO2* | NO | YES |
| *GIC2* | YES | YES |
| *RSN1* | NO | NO |
| *GYP8* | NO | YES |
| *MSB2* | NO | YES |
| *SCW10* | YES | YES |
| *AXL2* | NO | NO |

The promoter of each gene in the annotated Σ1278b genome was scanned for sequences matching the currently known Dig1 and Ste12 binding motifs. Yes/No indicates whether a motif is present as predicted by bioinformatics and represents the likelihood of regulation by the transcription factor. The p-values for enrichment of the Dig1 and Ste12 motifs are 1.33 e-9 and 1.43 e-9, respectively. Detailed motif scan results are shown in Supporting Dataset 2.
