# Supplementary material for: Control of Transcription by Cell Size
Source: PLoS Biol. 2010 Nov 2;8(11):e1000523. doi: 10.1371/journal.pbio.1000523 (PMC2970550; doi:10.1371/journal.pbio.1000523)
Supplement: Text S1 — Cell size mutants incompatible with cell size–transcription analysis. (0.03 MB DOC) [file pbio.1000523.s013.doc]

**Supporting Text 1.** Cell size mutants incompatible with size-transcription analysis.

We enlarged cell size of haploids using an ATP analog-sensitive allele of *CDC28* [17] that expands cell size upon inhibition by the analog [18]. This mutant showed an inverse correlation between *FLO11* expression and cell size that was consistent with our cell size-transcription hypothesis. However, under our experimental conditions, aberrant cell cycle progression and abnormal cell shape were observed in the presence of the analogue, precluding a clear interpretation of the results.

We treated a mid-log culture of asynchronously grown *cdc28*-as1 mutant with 50µM of the 1-NM-PP1(**9**) inhibitor and split the culture four ways: (1) no additional drug treatment (2) 0.1% azide (3) 10µM rapamycin (4) 100µg/mL cycloheximide. The PP1 treatment was intended to inhibit cell cycle progression while permitting cell growth, thereby enabling cells to enlarge. Azide, rapamycin or cycloheximide was added to inhibit cell growth independently of PP1 treatment. Samples were taken hourly in a 4-hour time course to track *FLO11* expression and cell size. Over time, cells treated with PP1 alone had significantly enlarged cell size and reduced *FLO11* expression compared to cells treated with additional growth inhibitors. However, we also found increasing expression of *CLN1* concurrent with the decreasing *FLO11* expression in the PP1 treated cells. Since expression of *FLO11* is repressed in the G1-stage of the cell cycle, we reasoned that the G1-like state in PP1 treated cells might repress *FLO11* independently of cell size.

We considered using PP1 at a lower concentration (500nM) to arrest cells in G2/M in the aforementioned experiment to avoid complications associated with G1. However, cells became hyperpolarized and thus had a fundamentally different morphology from that of WT cells, as shown in the original article by Bishop et al. [17]. Since the effect of cell shape on *FLO11* expression is also unclear, we would not be able to draw informative conclusions from this experiment.

We also considered releasing alpha-factor (G1) synchronized cells into PP1 to enlarge cell size, followed by washing away PP1 after different periods of incubation time to obtain cells of varying sizes in M-phase. However, we were not able to wash off PP1 efficiently, presumably due to the extraordinarily high affinity of PP1 for the Cdc28-as1 enzyme. As a result, the washed cells became hyperpolarized as described above.

The intrinsic temperature sensitivity of the Sigma 1278b strain background prohibited us from using temperature sensitive mutant alleles of cell cycle regulators to enlarge cell size as described [18].
