# Supplementary material for: Control of Transcription by Cell Size
Source: PLoS Biol. 2010 Nov 2;8(11):e1000523. doi: 10.1371/journal.pbio.1000523 (PMC2970550; doi:10.1371/journal.pbio.1000523)
Supplement: Text S2 — Discussion on genes up-regulated in large cells. (1.08 MB DOC) [file pbio.1000523.s014.doc]

**Supporting Text 2.** Discussion on genes up-regulated in large cells.

Genes up-regulated in large cells were significantly enriched for those containing the binding motifs of Ace2, Swi5, Rfx1 and Yap7 in their promoters (Supporting Dataset 2). Ace2 and Swi5 are similarly regulated in the cell cycle and are known to promote cytokinesis (separation between mother and daughter cells) [S1]. Rfx1 represses genes essential for DNA repair in the absence of DNA damage by recruiting the Tup1-Ssn6 transcriptional repressor complex to target genes [S2]. Yap7 is a transcription factor with a bZIP DNA-binding domain [S3] and unknown cellular functions. Ace2 and Swi5 appear to be the most relevant among these transcription factors, as genes promoting cytokinesis formed the only significant GO category for the up-regulated genes (Supporting Table 5). In addition, the up-regulation of these genes correlates with a significant phenotypic change: cytokinesis occurs more efficiently in the Σ1278b tetraploid than the haploid, making the tetraploid cells much less clumpy.

In contrast to pathways mediating transcriptional repression in large cells, pathways involved in differential induction by cell size are unclear. Ace2 and Swi5 are regulated by the cell cycle, but the cell cycle was not changed under our experimental conditions. First, the haploid size mutants were treated with nocodazole to minimize variations in cell cycle. In particular, the cell cycle arrest efficiencies of *bck2*∆ and *cln3*∆ were comparable to WT (Figure 3 and Supporting Table 6). Second, previous studies show that polyploidy does not have a noticeable effect on cell cycle progression [11, 15 in references for main text], and our RNA-seq results also did not show a change in cell cycle when we compared the haploid and tetraploid transcriptomes (Supporting Table 1).

Interestingly, the differential induction of cytokinesis-related genes only occurs in the Σ1278b strain background but not the S288c strain background (data not shown). This observation suggests that pathways differently regulated in the two strains are responsible for the up-regulation of gene expression in Σ1278b. Indeed, the Σ1278b haploid is significantly clumpier than S288c (see below), indicating that cytokinesis is less efficient in Σ1278b. This difference in “clumpiness” supports the notion that the two strains regulate differently the genes involved in mother-daughter separation.

**S288c cells are less clumpy than Σ1278b cells, suggesting strain-dependent differences in pathways regulating the separation of mother and daughter cells.**

| 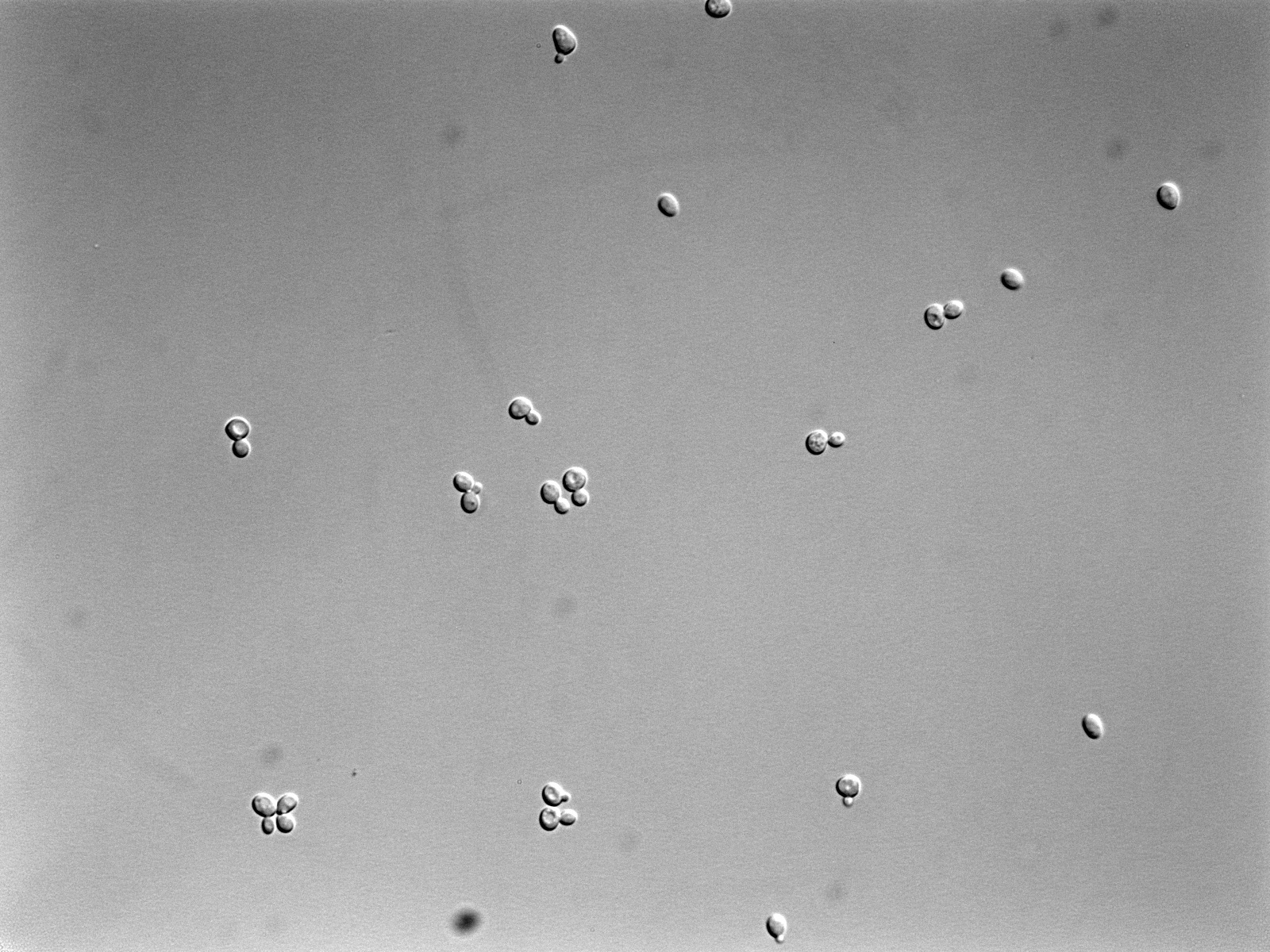  S288c haploid | 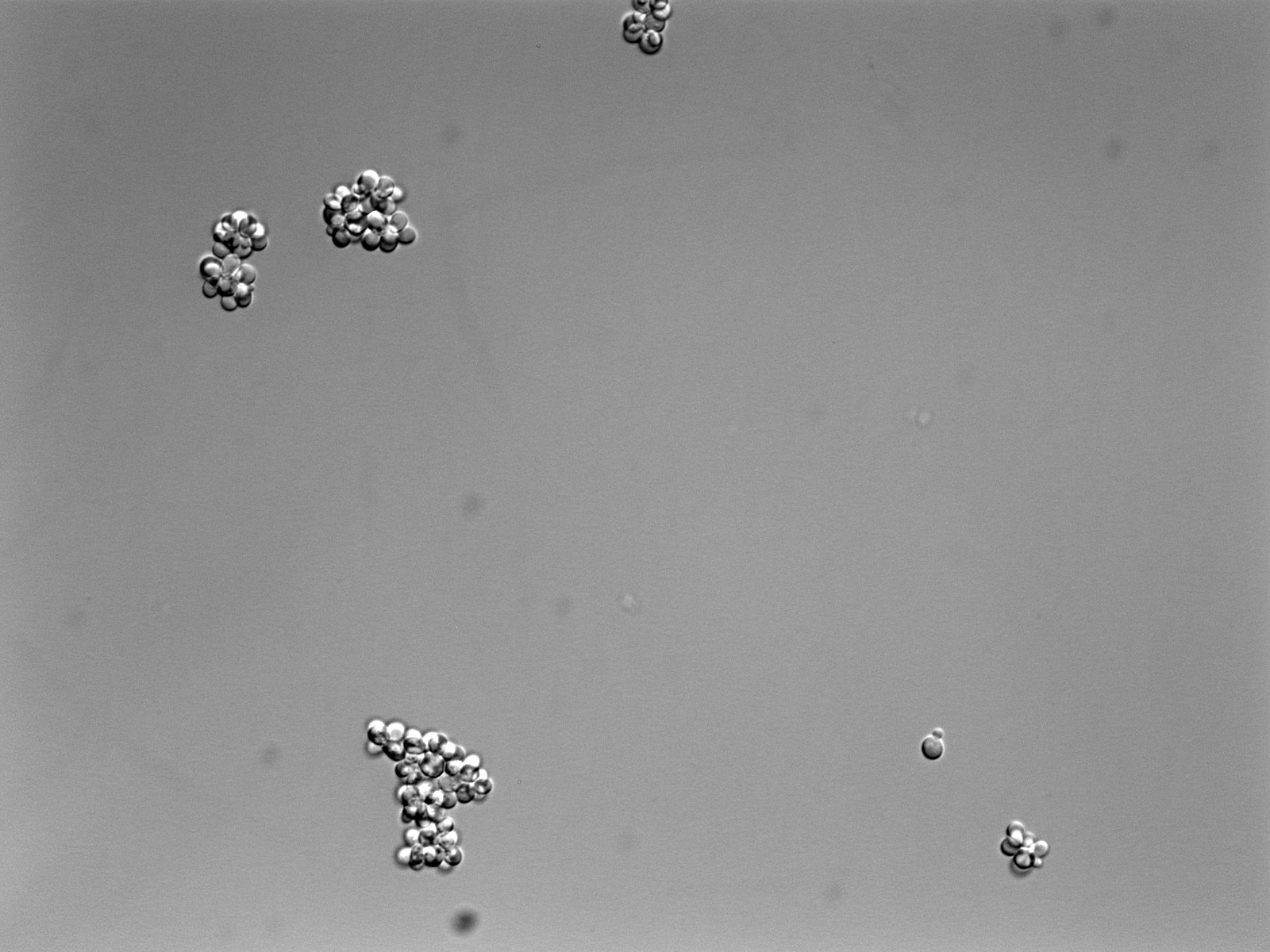  Σ1278b haploid |
| --- | --- |

Both strains are WT and *MAT***a**. The strains were grown in YPD at 30̊C until mid-log phase, and an aliquot of each culture suspension was imaged directly.

The clumpy phenotype of Σ1278b is not caused by a loss of function in enzymes that separate mother and daughter cells, but likely due to low expression levels of these enzymes. The Σ1278b tetraploid expresses elevated levels of these enzymes and is much less clumpy, indicating that the enzymes are functional.

References cited in this supporting document:

S1. Dohrmann PR, Butler G, Tamai K, Dorland S, Greene JR, et al. (1992) Parallel pathways of gene regulation: homologous regulators *SWI5* and *ACE2* differentially control transcription of HO and chitinase. Genes Dev 6: 93-104.

S2. Huang M, Zhou Z, Elledge SJ (1998) The DNA replication and damage checkpoint pathways induce transcription by inhibition of the Crt1 repressor. Cell 94: 595-605.

S3. Fernandes L, Rodrigues-Pousada C, Struhl K (1997) Yap, a novel family of eight bZIP proteins in *Saccharomyces cerevisiae* with distinct biological functions. Mol Cell Biol 17: 6982-6993.
